# *SLC6A4* STin2 VNTR genetic polymorphism is associated with tobacco use disorder, but not with successful smoking cessation or smoking characteristics: a case control study

**DOI:** 10.1186/1471-2156-15-78

**Published:** 2014-06-27

**Authors:** Márcia Regina Pizzo de Castro, Michael Maes, Roberta Losi Guembarovski, Carolina Batista Ariza, Edna Maria Vissoci Reiche, Heber Odebrecht Vargas, Mateus Medonça Vargas, Luiz Gustavo Piccoli de Melo, Seetal Dodd, Michael Berk, Maria Angelica Ehara Watanabe, Sandra Odebrecht Vargas Nunes

**Affiliations:** 1 Center of Approach and Treatment for Smokers, University Hospital, Londrina State University, Campus Universitário/Cx, Postal 600, Londrina, Paraná ZIP 86051-990, Brazil; 2 IMPACT Strategic Research Centre, School of Medicine, Deakin University, Geelong, Victoria, Australia; 3 Department of Psychiatry, Faculty of Medicine, Chulalongkorn University, Bangkok, Thailand; 4 Health Sciences Graduate Program, Health Sciences Center, State University of Londrina, Londrina, Brazil; 5 Department of Pathological Sciences, Biological Sciences Centre, Londrina State University, Londrina, Paraná, Brazil; 6 Department of Pathology, Clinical Analysis and Toxicology, Health Sciences Canter, Londrina State University, Londrina, Paraná, Brazil; 7 Orygen Youth Health Research Centre, Centre for Youth Mental Health, Department of Psychiatry and the Florey Institute for Neuroscience and Mental Health, University of Melbourne, Parkville, Australia; 8 Barwon Health and the Geelong Clinic, Swanston Centre, Geelong, Victoria 3220, Australia

**Keywords:** STin2 VNTR, Tobacco use disorder, Smoking cessation, Serotonin, Inflammation, Oxidative stress, Polymorphism, Genetic

## Abstract

**Background:**

The aim of this study was to determine if variable number of tandem repeats (VNTR) in the second intron (STin2) of the *serotonin transporter* (*SLC6A4*) gene was associated with tobacco use disorder, successful smoking cessation, or smoking characteristics. In this case–control study, patients with current tobacco use disorder, diagnosed according to DSM IV criteria (n = 185), and never-smokers, diagnosed according to CDC criteria (n = 175), were recruited and received 52 weeks of combined pharmacotherapy and cognitive therapy. Successful smoking cessation was defined as exhaled carbon monoxide < 6 ppm. *SLC6A4 gene* STin2 VNTR polymorphism was assessed using a Multiplex-PCR-based method. At baseline, participants were evaluated using the Fagerström Test for Nicotine Dependence (FTND) and the ASSIST scale.

**Results:**

The STin2.12 allele (OR = 2.45; 95% CI = 1.44-4.15, *p <* 0.001) was associated with an increased risk for tobacco use disorder, while the STin2.10/10 genotype (OR = 0.42; 95% CI 0.25-0.71, p < 0.001) decreased risk. There were no significant associations between tobacco use disorder and the STin2.10 or STin2.9 alleles or the other genotypes (STin2.12/12, 12/10, 12/9, 10/9 or 9/9). There were no significant associations between the STin2 genotypes and alleles and successful smoking cessation, smoking characteristics and increased alcohol or sedative use risk.

**Conclusions:**

Our results suggest that the STin2.10/10 genotype and STin2.12 allele are associated with tobacco use disorder or nicotine dependence, but not with treatment response or severity of dependence. It is hypothesized that the ST2in.12 allele by modulating the metabolism of serotonin may participate in the pathophysiology of tobacco use disorder or nicotine dependence.

## Background

Tobacco use disorder is a leading cause of mortality and disease burden [1,2]. Tobacco use disorder is a complex behavior that includes a number of stages of addiction, such as vulnerability to onset of use, continued use, propensity to become dependent and tobacco withdrawal [3-5]. 19% of ever smokers convert to daily smoking by the age of 15 years and 10% progress to smoking 20 cigarettes or more per day by the age of 18 [3]. Quitting smoking is beneficial to health at any age. Cigarette smokers who quit before age 35 years have mortality rates similar to those who never smoked. It is estimated that about 68.8% of adult smokers want to stop smoking, 52.4% attempted to quit in the past year, 6.2% had quit recently, 48.3% had been advised by a health professional to quit, and 31.7% had used counseling and/or medications when they tried to quit [4]. More than 80% of individuals who have tobacco use disorder attempt to quit smoking. 60% of the quitters, however, relapse within one week and less than 5% remain in sustained remission during a period of 12 months or longer.

Genetic factors and heritability contribute strongly to the onset of tobacco use and the development of tobacco use disorder [5]. Serotonin and the serotonin transporter (5-HTT) are implicated in the pathophysiology of tobacco use disorder [6]. The *SLC6A4* gene is located on chromosome 17 and three polymorphisms have been described: an insertion deletion in the promoter region, called 5-HTTLPR (serotonin transporter linked polymorphic region), a SNP G-T polymorphism in a non-coding 3 ′UTR, and the STin2 polymorphism, which is a 17 bp variable number of tandem repeat (VNTR) located in the second intron in *SLC6A4*[7]. The *SLC6A4* gene is the most frequently studied polymorphism in depression and tobacco use disorder [8,9]. The same gene may in part determine vulnerability for depression when exposed to multiple life stressors [10]. A study in 185 current smokers showed a positive association between neuroticism, an anxiety–related personality trait, and smoking behaviors and the S expression of the 5-HTTLPR region, but not the L genotype [11].

While there are now many reports on the association between 5-HTTLPR polymorphism of the *SLC6A4* gene and smoking behavior [11-25], there are only few studies on the *SLC6A4* gene STin2 polymorphism in tobacco use disorder [7,26]. The STin2 allelic variants were identified as 10-repeat and 12-repeat alleles that have been identified in all ethnicities, and the less common 9-repeat allele was only found in individuals of European or African descent [27]. An altered function of the STin 2 VNTR in the *SLC6A4* gene may be involved in tobacco use disorder since the STin2.12 allele has been reported to be a transcriptional enhancer associated with susceptibility to substance abuse [28]. It is now well established that nicotine increases serotonergic neurotransmission in the brain and symptoms of nicotine withdrawal may be mediated by a lowered serotonergic neurotransmission [7,29]. The STin2 polymorphism has also been associated with cognitive dysfunction in major depression [30].

Interestingly, the serotonin system and the *SLC6A4* gene have been implicated in the pathophysiology of psychiatric disorders which show a strong comorbidity with tobacco use disorder, including mood disorders and alcohol abuse [6,31]. Tobacco use and mood disorders are commonly comorbid conditions in patients of cigarette smoking cessation treatments [15,32-36]. In depressed smokers, depletion of serotonin in the brain is associated with a high risk for suicide and attempted suicide [35,37]. The short allele of 5-HTTLPR and the 12 repeat allele of STin2 are associated with a history of suicide attempts [38]. The serotonergic system has been associated with several personality traits that are related to an increased incidence of smoking, increased nicotine dependence, and difficulty in quitting smoking [39].

The aim of this paper was to delineate whether STin2 polymorphism of the *SLC6A4* gene is associated with a) tobacco use disorder, b) successful smoking cessation, c) smoking characteristics, including age at onset of tobacco use, duration of illness, lifetime cigarette consumption, years of smoking, severity of nicotine dependence, and d) comorbid substance use disorders, including alcohol and sedative abuse.

## Methods

### Cases and controls

In this case–control study, patients with current tobacco use disorder (n = 185) were recruited from outpatients at the Center of Approach and Treatment for Smokers, a smoking cessation program at Londrina State University (UEL), Paraná, Brazil. The controls were never-smokers (n = 175), recruited from staff at UEL. Patients with tobacco use disorder and never-smokers were men and women aged 18–65 and all ethnicities were accepted for this study. The diagnosis of tobacco use disorder was made by a senior psychiatrist using the semi-structured (SCID) interview translated into Portuguese [40]. In this study we only included current smokers who had smoked at least 100 cigarettes during their lifetime and, at the time of the interview, reported smoking every day or some days [41]. The controls, i.e. never-smokers, were subjects without tobacco use disorder who reported that they had never smoked a cigarette over their lifetime. Our never-smokers criteria are thus more stringent than the CDC criteria (41) for never-smokers, i.e. individuals who smoked less than 100 cigarettes in their lifetime. Cases with lifetime axis 1 diagnoses other than tobacco use disorder and affective disorders were excluded, including schizophrenia and psycho-organic syndromes. Patients with neuro-inflammatory and immune-inflammatory disorders were also excluded, including Parkinson’s disorder, stroke, multiple sclerosis, lupus erythematosus, rheumatoid arthritis, COPD, etc. The same exclusion criteria were applied to the never-smokers. The sample size was based on an a priori power calculation, which considered that with a power of 0.8, an effect size of 0.15 and α = 0.05 the total sample size should be around 350. A self-reported questionnaire was used to obtain information on socio-demographic characteristics, such as age, gender, marital status, ethnicity, years of education, and employment status. The study was conducted from March 2011 to July 2012. All subjects gave written informed consent to participate in the study after approval by the Ethics Research Committee at UEL, number 037/2011.

### Smoking characteristics

Smoking behavior was assessed through an interviewer-administered structured questionnaire. The Fagerström test for Nicotine Dependence (FTND) [42], translated and validated for use in Portuguese [43], was administered to all patients with tobacco use disorder. The FTND produces a score ranging from 0 to 10. Nicotine dependence was defined as a score ≥ 6 [44]. The number of pack-years was calculated as the number of cigarettes smoked per day multiplied by number of years smoked and divided by 20 (1 pack has 20 cigarettes).

Smoking status was also evaluated using exhaled carbon monoxide (CO_EXH_). CO_EXH_ was measured using a Micro CO Meter with an electrochemical sensor (Micro CO- Micro Medical Ltd, Rochester, Kent, UK). All participants were instructed to breathe deeply and to hold their breath for 20 seconds and then to exhale slowly and completely through a mouthpiece. The CO_EXH_ levels were dichotomized using 6 ppm as threshold value [45]. This threshold value was used as an additional inclusion criterion. Thus, never-smokers all had CO_EXH_ < 6 ppm, whereas those with current tobacco use disorder had a CO_EXH_ ≥ 6 ppm.

### Successful smoking cessation

All cases were treated for a period of 52 weeks with cognitive behavioral therapy sessions administered to groups of 10–15 participants and lasting for about 1½ hours. After the patient received an individualized assessment with the physician, he/she attends four weekly group sessions followed by two biweekly group sessions and then monthly sessions for a period of 52 weeks. Parallel to these group sessions, patients also receive pharmacological intervention, bupropion or nicotine replacement therapy, in accordance with the guidelines of the Ministry of Health, Brazil [46,47]. The combined program of tobacco use-focused cognitive therapy and pharmacological treatment is effective for both genders and depressed and non-depressed smokers [15]. Successful smoking cessation was assessed at the end of the treatment period as exhaled breath CO_EXH_ < 6 ppm. 64 of 185 subjects with tobacco use disorder were able to quit smoking during our 52 week treatment program.

### Substance use disorders

We used the Alcohol, Smoking and Substance Involvement Screening Test (ASSIST), which was developed by the World Health Organization, to screen levels of risk for alcohol and sedative use. We computed ASSIST scores for all participants. A risk score for alcohol was estimated as low risk (score 0–3), moderate risk (score 11–26) or high risk (score ≥ 27) and a risk score for sedatives was calculated as low risk (score 0–3), moderate risk (score 4–26) or high risk (score ≥ 27) [48]. The diagnoses of mood disorders, that is depressive disorder and bipolar disorder, was made by a trained psychiatrist using the semi-structured DSM-IV interview (SCID) using a validated Portuguese translation [40]. There were 112 individuals diagnosed with depression and 45 with bipolar disorder.

### Genotyping

Peripheral blood samples were obtained with EDTA as anticoagulant from all participants. Genomic DNA was extracted from 200 μL of peripheral blood cells using the Biopur Kit (Biometrix Diagnostic, Curitiba, Brazil) according to the manufacturer's instructions. The DNA pellet was re-suspended in 50 μL of Biopur Kit specific buffer, quantified by spectrophotometry, and stored in a -20°C freezer until use in genotyping analyses. Allelic Specific polymerase chain reaction (AS-PCR) for STin2 VNTR polymorphism detection were realized with genomic DNA (100 ng) with specific primers described by [48]. Forward primer — 5′TGGATTTCCTTCTCTCAGTGAATTGG3′ and Reverse primer —5′TCATGTTCCTAGTCTTACGCCAGTG3′. Samples were amplified using the kit buffer plus 1.25 units Taq polymerase (Invitrogen TM, Carlsbad, California). PCR conditions were: 5 min denaturation at 94°C, 40 cycles of 1 min at 94°C, 1 min at 60°C and 1 min at 72°C, and 20 min elongation at 72°C in a Master Cycler ( Eppendorf, Hamburg, Germany). Amplicons were analyzed by electrophoresis in 10% polyacrylamide gel and detected by a non radioisotopic technique using a commercially available silver staining method.

### Statistical analyses

The gene frequencies observed in patients with tobacco use disorder and never- smokers were compared using analyses of contingency tables (*χ*^2^ tests) with calculation of the Odds Ratios (OR) with a 95% confidence interval (CI). We used bivariate logistic regression analyses to assess the association between tobacco use disorder (with the controls as reference group) as dependent variable and the STin2 alleles and genotypes as explanatory variables, while controlling for the effects of other explanatory variables, including mood disorders, BMI, ethnicity, age, gender, years of education, etc. We used the logistic regression coefficients of the independent variables as estimators of the OR with 95% CIs. Relationships between the STin2 alleles and genotypes and continuous variables (e.g. age, years of education) were examined using analyses of variance (ANOVAs). Associations between diagnostic groups (cases versus controls) or gene frequencies and socio-demographic and clinical data were examined using contingency tables or Fisher exact probability test. Data have been expressed as mean ± standard deviation (SD). All the analyses were performed using SPSS (Version 20). A significance level of p-values ≤ 0.05 was used for statistical significance.

## Results

### Socio-demographic and clinical characteristics

Table 1 shows the socio-demographic and clinical characteristics of patients with current tobacco use (cases) and never-smokers (controls). No p-correction was employed to assess the results of multiple statistical univariate analyses carried out on the clinical and socio-demographic data because we used these results to delineate the relevant explanatory variables that were used as determinants of independent association with the diagnostic groups in multivariate analyses. Without p-correction, we found that there were significant differences in age between patients with tobacco use disorder and never-smokers. There were no significant differences in gender ratio or ethnicity between the two groups. Subjects with tobacco use disorder had a lower level of education than controls. In patients with tobacco use disorders there were more subjects who were unemployed or received disability support payments than in the control group. There were no differences in marital status and BMI between both groups. Patients with tobacco use disorder showed more mood disorders, and alcohol use and sedative use risk (and use of alcohol or sedatives) than never-smokers.

**Table 1:** Socio-demographic and clinical characteristics of patients with current tobacco use disorder (TUD) and never-smokers

**Variables**	**Smokers (n = 185)**	**Never-smokers (n = 175)**	***χ***^**2**^**/F/Ψ**	**df**	***p*** **value**
**Age (mean ± SD)**	48.7 (±10.5)	45.7 (±7.7)	9.78	1/358	p < 0.002
**Gender** Female/male	119/66	120/55	0.73	1	0.394
**Self-reported ethnicity**					
Caucasian	129	119	3.55	1	0.315
African	19	17			
Asian	5	12			
Others	32	27			
**Years of education**	9.64 (±5.38)	15.98 (±4.94)	135.78	1/358	p < 0. <0.001
**Employment status**					
Employed or student versus unemployed or disability support	155/30	175/0	0.293	-	<0.001
Stable relationship versus other					Pp0 0.109
**Marital status**	113/72	121/54	2.57	1	
**BMI (kg/m**^**2**^**)**	26.6 (±5.9)	26.5 (±4.2)	0.20	1/358	0.887
**Mood disorders** Yes/no	95/90	62/113	9.27	1	0.002
**ASSIST sedative use risk** Yes/no	14/171	0/175	0.196	-	<0.001
**ASSIST alcohol use risk** Yes/no	33/152	0/175	0.309	-	<0.001
**ASSIST ALL** Yes/no	44/141	0/175	0.363	-	<0.001

Results of analyses of variance (ANOVAS: age, BMI), *χ*^2^ test (ethnicity, gender, marital status, mood disorders) or Fisher exact probability test (employment status, all ASSIST ratings).

Results are shown as mean ± SD.

BMI: Body Mass Index.

ASSIST: Alcohol, Smoking and Substance Involvement Screening Test.

ASSIST ALL: sedative or alcohol use risk.

### Association between tobacco use disorder and STin2 alleles and genotypes

Table 2 shows the association between the Stin2 VNTR polymorphism and tobacco use disorder. The associations between tobacco use disorder and the 6 STin2 genotypes were tested at p = 0.0083 and those with the three STin2 alleles at p = 0.0166 (after p-correction was made for multiple comparisons). We found a significantly lower frequency of the STin2.10/10 genotype and a significantly higher frequency of the STin2.12 allele in patients with tobacco use disorder versus never-smokers. There were no significant differences in any of the other genotypes or for STin2.9 and STin2.10 alleles between cases and controls.

**Table 2:** STin2 VNTR polymorphism in patients with tobacco use disorder (TUD) versus never-smokers

**STin2 VNTR**		**Never-smokers n = 175**		**TUD n = 185**		**OR**	**95% CI**	***χ***^**2**^	***p***
		**Yes**	**No**	**Yes**	**No**				
Genotypes	STin2 - 12/12	72	103	82	103	0.88	0.58-1.33	0.54	0.372
	STin2 - 12/10	50	125	71	114	1.56	1.00-2.42	3.88	0.049
	STin2 - 12/9	3	172	6	179	0.63	0.15-2.67	0.72	0.353
	STin2 - 10/10	49	126	26	159	0.42	0.25-0.71	10.60	< 0.001
	STin2 - 10/9	0	175	0	185	*-*	*-*	-	-
	STin2 - 9/9	1	174	0	185	-	-	-	-
Allelic Variants	STin2.12	125	50	159	26	2.45	1.44-4.15	11.38	< 0.001
	STin2.10	99	76	97	88	0.85	0.56-1.28	0.621	0.431
	STin2.9*	4	171	6	179	1.43	0.40-5.17	-	0.751

*OR:* odds ratio with 95% CI: confidence interval). All results of *χ*^2^ tests (df = 1), except * result of Fisher exact probability test.

Table 3 shows the results of logistic regression analyses with tobacco use disorder as dependent variable (and controls as reference group) and the STin2.12 allele and STin2.10/10 genotype, age, gender, education, mood disorders, as explanatory variables. We found that the STin2.12 allele, the diagnosis of mood disorders and years of education predicted the incidence of tobacco use disorder versus never-smokers (*χ*2 = 141.61, df = 3, p < 0.001; Nagelkerke = 0.43). Forced entry of additional explanatory variables showed no significant effect of age (Wald = 1.91, df = 1, p = 0.167), gender (Wald = 1.24, df = 1, p = 0.266), self-reported ethnicity (Wald = 5.00, df = 1, p = 0.288), marital status (Wald = 2.21, df = 3, p = 0.529) and BMI (Wald = 1.58, df = 1, p = 0.209). There was a marginal, but significant effect of employment status (Wald = 4.01, df = 1, p = 0.045). We found that the STin2.10/10 genotype, mood disorders and years of education were associated with the incidence of tobacco use disorder versus never-smokers (*χ*^2^ = 141.64, df = 3, p < 0.001; Nagelkerke = 0.43). Forced entry of additional explanatory variables showed no significant effect of age, gender, self-reported ethnicity, marital status and BMI. There was a marginal but significant association between tobacco use disorder and employment status (Wald = 5.41, df = 1, p = 0.020). Thus, adjusting for additional relevant explanatory variables, including mood disorders, did not change the associations between tobacco use disorder and STin2 alleles and genotypes, and revealed that mood disorders and years of educations were significant predictors.

**Table 3:** Results of automatic (step-up) binary logistic regression analyses with tobacco use disorder (TUD) as dependent variable (never-smokers as reference group) and the STin2.12 allele (regression 1) or STin.10/10 genotype (regression 2) and the other listed variables as explanatory variables

	**Variables**	**Wald**	**df**	**p value**	**OR**	**95% CI**
Regression 1	STin2.12 allele	8.68	1	0.003	2.68	1.39 – 5.18
	Mood disorders	6.51	1	0.011	1.97	1.17 – 3.32
	Years of education	72.98	1	<0.001	0.76	0.71 – 0.81
Regression 2	STin2.10/10 genotype	8.14	1	0.004	0.38	0.20 – 0.74
	Mood disorders	6.64	1	0.010	1.98	1.18 – 3.34
	Years of education	72.99	1	<0.001	0.76	0.71 – 0.81

OR: odds ratio with 95% CI: 95% confidence interval.

### Association between smoking characteristics and STin2 alleles and genotypes

We have also computed whether, in patients with tobacco use disorder, there were associations between STin2 VNTR genotypes and alleles and successful smoking cessation 52 weeks after starting treatment and smoking characteristics, i.e. age at onset of tobacco use disorder, duration of illness, cigarettes/day and pack/years, the FNDS score and attempts to quit smoking. Table 4 shows the associations between the STin2 polymorphism and smoking cessation and smoking characteristics. Even at very liberal p-values (p-correction for multiple comparisons) of p = 0.0083 (for the genotypes) and p = 0.0166 (for the alleles) we were unable to find any significant associations between the STin2 VNTR genotypes and alleles and successful smoking cessation and clinical smoking characteristics.

**Table 4:** Associations between tobacco use disorder (TUD) characteristics and STin2 VNTR genotypes and allelic variants

**Smoking parameters**		**Genotypes**				**Allelic variants**	
			**STin2 10/10**	**STin2 12/10**	**STin2 12/12**	**STin2.12**	**STin2.10**
		**n**	P*	P*	P*	P*	P*
Onset of TUD (years)	14.81 (±3.91)	185	0.868	0.170	0.156	0.868	0.146
Duration of TUD (years)	33.59 (±11.28)	185	0.061	0.262	0.478	0.061	0.837
Cigarettes/day	22.28 (±13.45)	185	0.120	0.987	0.377	0.120	0.274
Pack-years	37.02 (±28.11)	185	0.017	0.617	0.239	0.017	0.247
Fagerström score	5.71 (±2.21)	185	0.361	0.670	0.591	0.361	0826
Attempts at smoking cessation	1, 2 or 3 attempts	86/67/32	0.948	0.852	0.728	0.948	0.739
Successful smoking cessation	Yes/no	64/185	0.658	0.858	0.612	0.658	0.630

Results are shown as mean ± SD.

*p values obtained in analyses of variance (all df = 1/183) or *χ*^2^ tests (all df = 1).

### Comorbidities with substance use risk

Table 1 shows that participants with tobacco use disorder had significantly higher scores on the sedative and alcohol ASSIST scales than participants without tobacco use disorder. Nevertheless, we could not find any relationships between the STin2.12 allele or the STin2.10/10 genotype and the ASSIST scale measures. There was no significant association between the STin2.12 allele and alcohol use risk (29/255 versus 4/72, *χ*^2^ = 1.76, df = 1, p = 0.184), risk for sedative use (11/273 versus 3/73, *χ*^2^ = 0.00, df = 1, p = 0.976) or either sedative or alcohol use risk (37/247 versus 7/69, *χ*^2^ = 0.81, df = 1, p = 0.367). There was no significant association between the STin2.10/10 genotype and sedative use risk (4/71 versus 29/256, *χ*^2^ = 1.67, df = 1, p = 0.196), risk for sedative use (3/72 versus 11/274, *χ*^2^ = 0.00, df = 1, p = 0.955) or either sedative or alcohol use risk (7/68 versus 37/248, *χ*^2^ = 0.81, df = 1, p = 0.391).

## Discussion

The major finding of this study is that the Stin2.10/10 genotype decreased risk whereas the STin2.12 allele increased risk to tobacco use disorder. Our results are in agreement with a previous report showing that carrying the STin2.10 allele was more common in non-smokers compared with smokers, showing a protective effect of this allele [7]. Our results also extend previous findings on a “significant excess of the 5-HTTLPR long allele with the 12-repeat VNTR in smokers” [26]. In another study, it was found that allele 10 carriers were less prevalent in smokers than in non-smokers, indicating a protective effect of the STin2.10 allele [7]. Our results are not in agreement with those of Alves de Lima et al. [7] who found that subjects carrying STin2. 9 allele carriers were more prevalent in smokers than in non-smokers. These contradictory results may be explained by differences in study populations. Thus while our study and that of Alves de Lima et al. [7] were both performed in a Brazilian population, the latter authors examined smokers with and without cancer, whereas in our study no cancer patients were included but instead more subjects with affective disorders.

In our study we found that patients with current tobacco use disorder showed a significantly increased prevalence of mood disorders, more work related disability and a lower education level than never-smokers. These results are consistent with previous reports which showed that current smoking is associated with subsequent depressive disorders, increased work disability and lower education levels [32,34,35,41]. Lower educational levels are additionally associated with the initiation of tobacco use disorder and with an increased risk to be unable to quit smoking [5]. Nevertheless, even after considering the effects of mood disorders and years of education the association between tobacco use disorder and the ST2in polymorphism remained significant.

The second major finding of this study is that there were no significant associations between the STin2 alleles or genotypes and either successful smoking cessation at week 52 or smoking characteristics, such as age at onset, duration of tobacco smoking, severity of tobacco smoking, number of cigarettes/day or packs/year etc. These negative findings extend those of a previous study showing that the *SLC6A4* gene is not a major determinant associated with attempts to quit smoking [19]. As Kremer et al. [26] we detected a highly significant association between the 5-HTT and the case-definitions of tobacco use disorder (in our study) or smoking (in Kremer’s study), but not with dependency levels or smoking characteristics. Therefore, we may conclude as Kremer et al. [26] that this polymorphism influences the pathogenesis of tobacco use disorder or nicotine dependence. Other studies showed that other genes related to tobacco use disorder may also modulate cessation attempts. Thus, one study tested the effects of genetic risk in a cohort that initiated smoking during adolescence progressed to daily smoking and progressed to heavy smokers and developed nicotine dependence. The authors examined the effects of the SNP of the q 25.1 region of chromosome 15 containing the nicotinic cholinergic receptor CHRNA5, CHRNA3, CHRNB4 gene cluster. Genetic risk score was related to individuals who were more likely to develop nicotine dependence and were more likely to fail in their cessation attempts [3]. In addition, serum cotinine levels were associated with a CHRNA genetic polymorphism [49]. Genome wide association studies of tobacco addiction have also identified genes that affect smoking initiation, these genes being associated with a brain-derived neurotrophic factor (BDNF) polymorphism on chromosome 11 [50]. These findings are consistent with the idea that different genes are associated with the development and progression of smoking behavior from initiation, nicotine dependence, daily smoking to smoking cessation.

The results of this study add to the knowledge that tobacco use disorder is a complex behavior that includes polygenic risk. Several regions across the genome have been implicated in containing genes that confer liability to tobacco use disorder or nicotine dependence and variation in individual genes has been associated with nicotine dependence. Regarding the interplay between genetic and environmental influence on the etiology of nicotine dependence, studies of twins found that 50% of the risk of nicotine dependencies was genetically transmitted [51]. More specifically, the STin2.12 allele may be a transcriptional enhancer associated with an increased susceptibility to substance abuse [28]. There is evidence that the STin2.12 allele may have a higher transcriptional activity than the 10-repeat allele [38] and that STin2.12 allele homozygotes show lowered serotonin availability [30]. Nicotine is known to increase the release and signaling of serotonin [52,53]. This may suggest that disorders in 5-HTT functioning and 5-HT signaling may play a role in nicotine dependence or withdrawal [52]. Antidepressants, such as selective serotonin reuptake inhibitors, have, however no efficacy in quitting smoking [54]. In a brain imaging study, there were no associations between STin.2 genetic polymorphism and the availability of the 5-HTT in different brain regions [55].

Nevertheless, smoking causes activated immune-inflammatory and oxidative and nitrosative stress (IO&NS) pathways [15,34-36]. Activated IO&NS pathways, in turn, may induce indoleamine 2,3-dioxygenase (IDO) leading to increased levels of tryptophan catabolites (TRYCATs), including kynurenine [56], and lowered levels of tryptophan and thus serotonin [37]. Therefore the lowered availability of serotonin associated with the STin2.12 allele may be aggravated by smoking-induced IDO activation. Such IO&NS and IDO responses are strongly related to depression and depressive symptoms in patients with tobacco use disorders [56,57]. Nicotine abuse may then be regarded as an operationally conditioned response that counteracts depleted serotonin levels thus preventing the adverse effects of lowered serotonin. Smoking-induced activation of IO&NS pathways may further endanger serotonin metabolism thereby maintaining nicotine abuse and thus tobacco use disorder. Therefore, it is likely that the 5-HTT genes may contribute to the development of tobacco use disorder or nicotine dependence among individuals who are prone to mood disorders or have a lower educational level. In addition, the effects of nicotine use on serotonin, and smoking- and STin2-related changes in the IO&NS-serotonin nexus may activate (neuro)degenerative pathways related to dysfunctions in the hypothalamic-pituitary-adrenal (HPA) axis, microglial activation, mitochondrial dysfunctions, decreased levels of antioxidants, damage to lipids, proteins, and DNA leading to autoimmune responses against multiple neoantigens [56,57]. Future research should examine the relationships between IO&NS pathways and serotonin signaling in tobacco use disorder and nicotine-dependence.

A third major finding is that no significant association could be established between STin2 polymorphism and alcohol use and sedative use risk. Nevertheless, STin2 polymorphism was associated with tobacco use disorder and tobacco use disorder with increased alcohol and sedative use risk. This may indicate that the STin 2 VNTR polymorphism in the *SLC6A4* gene could influence an individual’s vulnerability to develop tobacco use disorder rather than a substance use disorder. Phrased differently, the STin2.12 allele and STin2.10/10 genotype may be specifically associated with tobacco use disorder. However, we used the ASSIST scale to measure increased risk to alcohol and sedative use rather than DSM IV diagnostic criteria and therefore the results should be checked using DSMV criteria of substance abuse disorder.

The results of this study should be interpreted with regard to its strengths and limitations. Firstly, the present study design was a case–control study and therefore our results can only delineate associations and not causality. Secondly, our sample included smokers who had sought smoking cessation treatment, while women are more likely to seek assistance for smoking cessation than men. Therefore, our sample may not be representative of the general population. Thirdly, the age of our sample ranged from 18 to 65 years old and therefore our findings cannot be generalized to older or younger population. Fourthly, in this study we did not examine other polymorphisms in the *SLC6A4* gene.

## Conclusions

Our findings provide some evidence that a lower frequency of the STin2.10/10 genotype and a higher frequency of the STin2.12 allele are more frequent among individuals with current tobacco use disorder than in never-smokers, suggesting that this 5-HTT polymorphism is related to the serotonergic pathophysiology of tobacco use disorder and nicotine dependence and the consequences of smoking activating IO&NS pathways. The 5-HTT polymorphism does not appear to be a major determinant of smoking cessation or smoking characteristics, suggesting that this polymorphism is related to the pathogenesis of tobacco use disorder or nicotine dependence. This 5-HTT polymorphism may be more specific to tobacco use disorder rather than to substance abuse disorder. The translational implications of these findings include the identification of subgroups of patients with current tobacco use disorder, for example, those with a serotonergic pathophysiology and those who are more at risk to develop mood disorders. Elucidating the influence of the 5-HTT gene polymorphism is important among patients with current tobacco use disorder because smoking may reinforce the dysfunctions in serotonergic signaling through induction of IO&NS pathways.

## Competing interests

The authors declare that they have no competing interests.

## Authors’ contributions

All authors participated in its design, reviewed drafts of the manuscript and approved the final version before submitting for publication.
